# Attraction and disruption: how loop extrusion and compartmentalisation shape the nuclear genome

**DOI:** 10.1016/j.gde.2024.102194

**Published:** 2024-06

**Authors:** Mikhail Magnitov, Elzo de Wit

**Affiliations:** Division of Gene Regulation, the Netherlands Cancer Institute, Plesmanlaan 121, 1066 CX Amsterdam, the Netherlands

## Abstract

Chromatin loops, which bring two distal loci of the same chromosome into close physical proximity, are the ubiquitous units of the three-dimensional genome. Recent advances in understanding the spatial organisation of chromatin suggest that several distinct mechanisms control chromatin interactions, such as loop extrusion by cohesin complexes, compartmentalisation by phase separation, direct protein–protein interactions and others. Here, we review different types of chromatin loops and highlight the factors and processes involved in their regulation. We discuss how loop extrusion and compartmentalisation shape chromatin interactions and how these two processes can either positively or negatively influence each other.

**Current Opinion in Genetics & Development** 2024, **86**:102194

This review comes from a themed issue on **Genome Architecture and Expression**

Edited by **Maya Capelson** and **Eda Yildirim**

For complete overview of the section, please refer to the article collection, “Genome Architecture and Expression (2024)”

https://doi.org/10.1016/j.gde.2024.102194

0959–437X/© 2024 The Author(s). Published by Elsevier Ltd. This is an open access article under the CC BY license (http://creativecommons.org/licenses/by/4.0/).

## Introduction

When stretched out, the mammalian genome is about 2 metres long, yet it has to fit into a nucleus that is only a few micrometres in diameter. To achieve this, chromatin, made up of DNA and its associated proteins, must be tightly packed and organised into a complex spatial structure. This structure is shaped in a nonrandom manner to guide and ensure proper gene expression, DNA damage repair, DNA replication and other processes [1]. The chromatin loop is the core unit of spatial genome organisation and can be defined as two loci on the same chromosome that are in close physical proximity. In this review, we discuss recent advances in the mechanisms and factors that mediate preferential interactions between different classes of chromatin regions in the nuclei of vertebrate organisms, excluding the organisation of interphase genomes in less complex organisms, which is reviewed elsewhere 2, 3, 4.

## Extrusion-mediated loops

One of the major processes shaping the organisation of DNA in the nucleus is loop extrusion, in which initially small DNA loops are progressively enlarged by DNA motors known as structural maintenance of chromosomes (SMC) protein complexes. The cohesin complex, one of the SMC complexes, is involved in chromatin looping during interphase [5]. After loading onto chromatin, cohesin facilitates ATP-dependent DNA extrusion and loop expansion stimulated by nipped-B-like protein (NIPBL). Cohesin is released from chromatin by wings apart-like protein homolog (WAPL), freeing the complex to initiate a new cycle of extrusion, highlighting the dynamic nature of the process [5]. The rate of extrusion, and therefore which distal loci are brought together, depends on the composition of the cohesin complex, the acetylation of its subunits and the binding of cofactors, which has been reviewed in detail elsewhere 1, 5, 6. In addition, DNA-binding proteins and other chromatin-based barriers can block extrusion, resulting in the formation of specific cohesin-dependent loop types.

### Barriers to cohesin-mediated loop extrusion

**Figure 1:**
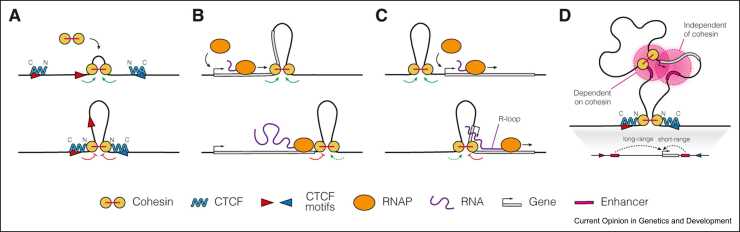
Models of extrusion-mediated interactions and barriers to extrusion. **(A)** CTCF anchors cohesin-mediated loop extrusion. Cohesin recruitment to chromatin forms a small nascent loop upon loading. Once loaded, cohesin facilitates bidirectional DNA extrusion and loop expansion until it encounters CTCF bound to DNA with its N-terminus facing cohesin. Convergently oriented CTCF molecules are thus able to form a loop. **(B)** RNAPII counteracts loop extrusion by translocating and displacing cohesin. In a head-on collision with RNAPII, cohesin is translocated towards the 3′ end of the gene. Cohesin-mediated extrusion on the other side of the collision may remain unaffected. RNAPII pushes cohesin to the end of its trajectory at the TTS or until cohesin bypasses RNAPII. **(C)** R-loops act as cohesin barriers at active genes and enhancers. Upon codirectional collision with the transcription process, R-loops or RNAPII itself can slow down loop extrusion in one direction. **(D)** Loop extrusion is involved in E–P interactions. The degree of cohesin dependence for a gene depends largely on the distance to the enhancer. In the case of the distal enhancer, cohesin may be involved in E–P loop formation by bringing the enhancer and promoter closer together. However, loop extrusion may not be essential for the formation of the short-range E–P contact. In addition, CTCF-anchored loops may limit enhancer action to prevent the formation of ectopic E–P loops.

The most prominent extrusion barrier is the DNA-binding protein CCCTC-binding factor (CTCF). CTCF anchors cohesin via its N-terminus, which directly interacts with cohesin and is essential for its retention 7, 8, 9. This interaction is mediated by two aromatic amino acid residues in CTCF, the mutation of which disrupts the cohesin-CTCF interaction, resulting in the complete loss of CTCF-anchored chromatin loops [9]. The orientation of the CTCF molecule binding to DNA depends on the orientation of its nonpalindromic motif. Sites that orient the N-terminus of CTCF towards the extruding cohesin give rise to the loop anchors (Figure 1A). Therefore, the majority of CTCF-anchored loops are formed between sites with convergently oriented CTCF motifs. Manipulation of CTCF motifs or CTCF abundance disrupts the loops and reduces interactions between distal CTCF loci 10, 11. The ability of CTCF to block cohesin has been demonstrated *in vitro* 12, 13••. A recent study suggests that CTCF modulates the behaviour of cohesin-mediated extrusion in a DNA tension–dependent manner [13]. As DNA tension increases, CTCF changes its mode of action from turning cohesin into a one-sided extruder to completely blocking the extrusion process and even inducing loop shrinkage [13]. This raises the possibility that processes that alter DNA tension by promoting either supercoiling or unwinding may also contribute to the regulation of CTCF-anchored loops.

The dynamics of loop extrusion around active genes can be modulated by the transcription process. While some studies have suggested a limited role for transcription in chromatin looping 14, 15, more recent studies have shown that the depletion of RNA polymerase II (RNAPII) leads to longer chromatin loops [16] and rewired interactions between CTCF sites [17]. Although the effects are relatively mild and secondary effects following depletion cannot be excluded, these results suggest that transcription can counteract and constrain cohesin-mediated loop extrusion. Additional evidence that RNAPII repositions cohesin comes from several studies reporting the accumulation of cohesin at transcription termination sites (TTS), where it is presumably repositioned by RNAPII (Figure 1B), although these observations were made in cells lacking both CTCF and WAPL 18, 19, 20. In addition, it has been shown that read-through transcription by RNAPII following *in vitro* infection of monocytes with influenza can displace cohesin from CTCF sites proximal to the TTS, thereby eliminating specific CTCF-anchored loops [21]. Inhibition of transcription elongation was able to rescue such cohesin displacement and restore disrupted interactions. Transcription start sites of active genes have also been shown to accumulate cohesin [19]. However, it is unclear how cohesin is stalled at these sites without being apparently pushed by RNAPII. Although RNAPII may act as a barrier in this case, it extends in the same direction as extrusion, raising the possibility that additional mechanisms may be involved. One such mechanism could be R-loops [12], which have recently been described as cohesin barriers and could contribute to cohesin stalling at active genes and possibly enhancers (Figure 1C).

### Interplay of loop extrusion and its barriers

Loop extrusion also plays an important role in the formation of enhancer–promoter (E–P) loops, and both CTCF and RNAPII contribute to this process. CTCF-anchored loops can either bridge interactions between promoter-proximal and enhancer-flanking CTCF sites or increase the overall contact probability to facilitate E–P interactions independently of cohesin stalling 15, 22, 23•, 24, 25. Conversely, consistent with the original definition of CTCF as an insulator protein, cohesin-blocking activity at CTCF-bound sites may also prevent the formation of aberrant E–P loops 22, 26. On the other hand, RNAPII can directly anchor and stabilise E–P loops 17•, 27 and contribute to cohesin pausing 19, 20.

What could be the role of loop extrusion in E–P communication? Studies in both native and engineered regulatory landscapes have shown that E–P interactions of ∼50–100 kb or longer require cohesin, whereas those of ∼10–50 kb do not, although the exact distances vary depending on the cell type investigated 23•, 28, 29 (Figure 1D). Although there is increasing evidence that loop extrusion contributes to E–P communication and that cohesin is associated with promoters and enhancers, whether cohesin is directly loaded there or is stalled after extrusion remains unclear and requires further investigation 19, 22, 30, 31, 32, 33. It is important to note that recent results suggest that cohesin may be transiently dispensable for most E–P loops 34, 35, 36, 37, 38••. It is therefore plausible that although cohesin-mediated loop extrusion is required to form E–P loops, there may be additional mechanisms that function independently of cohesin to maintain these interactions.

## Compartmentalisation-mediated loops

Chromatin compartmentalisation is the spatial separation of multiple sequentially arranged chromatin regions with similar biochemical properties, also known as block copolymers. It is driven by a physical process known as microphase separation, which is facilitated by interactions between polymeric regions of the same type. These interactions can be achieved by either polymer–polymer phase separation (PPPS) or liquid–liquid phase separation (LLPS) [39]. PPPS is mediated by factors that can directly bridge two sections of a chromatin polymer. LLPS occurs when chromatin regions act as scaffolds, generating a high local concentration of chromatin-associated factors. Many of these proteins have intrinsically disordered regions (IDRs), and the weak attractions between them can promote the formation of condensates that link distal chromatin polymer sections [40]. Compartmentalisation thus mediates different chromatin loop types over a wide range of genomic distances and scales, depending on the scaffold region and the chromatin-binding proteins.

### Separation of inactive heterochromatic regions

**Figure 2:**
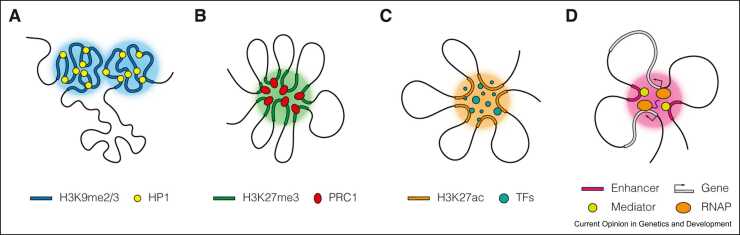
Models of compartmentalisation-mediated loops. **(A)** HP1 binds to H3K9me2/3 histone modifications and promotes interactions between distal heterochromatic regions through both dimerisation and phase separation mechanisms. These interactions spatially segregate the genome into active A and inactive B compartments. **(B)** PcG proteins cluster H3K27me3-rich regions and facilitate Polycomb loop formation. Polycomb loops are likely driven by phase separation of PRC1 subunits. Polycomb subcompartments are associated with a repressed chromatin environment. **(C)** TFs and chromatin regulators contribute to interactions between distal CREs. Their IDR-mediated phase separation capabilities, promoted by acetylated chromatin, in particular the H3K27ac mark, facilitate looping between superenhancers and thus contribute to the formation of the superenhancer subcompartment. **(D)** E–P interactions are organised into nested microcompartments. Since phase-separating factors are abundant at both enhancers and promoters, microcompartments may be the result of phase separation of RNAPII and Mediator and bridging interactions between TFs. Together, these factors sustain E–P loops in a potentially redundant manner.

The most prominent example of compartmentalisation shaping the genome is the spatial segregation of euchromatic and heterochromatic regions, termed A and B compartments, respectively. Segregation of the A and B compartments is proposed to be driven by preferential attraction between constitutive heterochromatic regions [41]. The attraction between these regions is tightly linked to H3K9me3 histone modification presence 42, 43, 44, 45 and is thought to be facilitated by heterochromatin protein 1 (HP1), which binds to H3K9me2/3 histone marks (Figure 2A). HP1 has been shown to bridge H3K9me3-containing nucleosomes by dimerisation [46], but also to undergo phase separation with H3K9me3 nucleosomal arrays and its binding partners SUV39H1 and TRIM28 *in vitro* 47, 48, 49. However, the behaviour of HP1-mediated heterochromatin in mouse embryonic fibroblasts does not appear to exhibit phase separation properties [50]. Although HP1 has been implicated in the establishment of heterochromatin clustering in *Neurospora* and *Drosophila* 51, 52, the causal role of HP1 in this process remains to be determined. Therefore, the mechanisms by which HP1 is involved in heterochromatin formation in living cells are not fully elucidated and may entail both phase separation and bridging of heterochromatic regions, as well as additional factors or mechanisms [53].

Another type of distal interactions within heterochromatin regions is mediated by Polycomb group (PcG) proteins, which have long been implicated in chromatin looping, promoting interactions between distal H3K27me3-rich regions that are generally silent 54, 55, 56. It has been shown that tethering PcG proteins to an artificial site in the genome is sufficient to induce distal interactions between Polycomb-bound sites 57, 58. Polycomb repressive complex 1 (PRC1) recruitment to chromatin is facilitated by chromobox protein homolog 2 (CBX2), a reader of the histone modification H3K27me3. CBX2 has been shown to undergo phase separation through its IDR domain, leading to the formation of Polycomb condensates [59]. Several other subunits of the PRC1 complex have also been shown to contribute to Polycomb condensate formation through heterotypic interactions [60]. Recently, deletion of the PRC1 core subunit RING1B resulted in reduced interactions between PRC1-bound genomic regions, but impairment of catalytic activity by mutation of RING1B had no effect on these loops 61, 62. This suggests that the presence of the PRC1 complex, but not its enzymatic activity, is required for Polycomb loop formation (Figure 2B).

### Interaction network of regulatory elements

*Cis*-regulatory elements (CREs) can also form clusters in the nucleus. Such behaviour has been observed for superenhancers 63, 64, enhancer elements bound by pluripotency-associated transcription factors (TFs) 55, 65, and, more recently, for virtually all CREs [66]. These interactions occur over distances of tens of megabases and can even occur *in trans*^63^, ^64^, ^66^, creating a highly interconnected subcompartment that can enhance target gene activation even when the CRE is repositioned [67]. Chromatin at CREs is often highly acetylated and bound by transcriptional regulators, many of which have IDRs. This could in turn drive the formation of long-range chromatin loops as a consequence of phase separation 68, 69, 70, 71 (Figure 2C). The bromodomain and extraterminal protein BRD4 and the transcription factor CEBPA have been shown to promote looping interactions that are attenuated in their absence 70, 72. Another way of promoting looping of CREs is by factors that bind active chromatin and drive its compartmentalisation, as recently shown for BRD2, although the exact mechanism of this process is unknown [73]. It is important to emphasise that the disruption of enhancers *per se* may result in reduced chromatin looping, and therefore, additional experiments demonstrating that BRD2 and BRD4 are sufficient to drive CRE interactions are required, along with a more detailed exploration of the underlying mechanisms. Thus, the full complement of enhancer-associated proteins capable of promoting CRE looping remains to be determined.

Recently, high-resolution chromosome conformation capture approaches have revealed that E–P loops are organised into nested focal interactions 38••, 74. As the structure of these interactions resembles compartments at a smaller scale, these E–P interactions have been termed microcompartments, and it has been proposed that a compartmentalisation mechanism may contribute to their formation (Figure 2D). Indeed, several components involved in E–P interactions are capable of phase separation. First, RNAPII clustering and chromatin association at active promoters have been shown to be mediated by phase separation of its carboxyterminal domain. While some E–P loops were found to be affected by transcriptional inhibition 27, 38••, 74, the majority were maintained, suggesting that the presence of RNAPII, rather than its action, may be more important in establishing these interactions. Indeed, RNAPII depletion leads to a genome-wide reduction in E–P interactions, although it does not completely abolish them [17]. Second, the Mediator complex, a key component of the transcriptional regulatory machinery, can phase separate and form condensates with many TFs [68]. Whether Mediator directly bridges enhancers and promoters is unclear 75, 76; however, it may cooperate closely with enhancer-bound TFs and promoter-bound RNAPIIs to facilitate E–P looping. Finally, bridging interactions through oligomerization of TFs and co-regulators, such as GAGA in *Drosophila* or LDB1 in humans, can play a role in E–P interaction 77, 78, 79. It should be noted that cooperation between chromatin factors and redundancy in their phase separation capabilities may explain why depletion of a single factor does not drastically affect or completely abolish E–P loops.

## Conclusions and future perspectives

**Figure 3:**
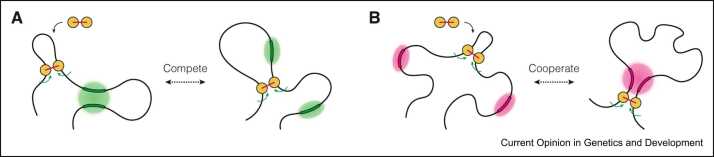
Models of antagonistic and agonistic relationships between loop extrusion and compartmentalisation mechanisms. **(A)** Loop extrusion disrupts loops by perturbing the compartmentalised chromatin environment. **(B)** Loop extrusion facilitates loop formation by aiding the process of compartmentalisation.

Both loop extrusion and compartmentalisation form chromatin loops and contribute to genome organisation in the nucleus. They are two distinct physical mechanisms, and the mechanical force exerted by loop extrusion has the propensity to perturb or enhance weak interactions mediated by compartmentalisation (Figure 3). In the case of Polycomb loops, cohesin-mediated loop extrusion acts antagonistically to disrupt these looping interactions [80]. Interestingly, in the case of super-enhancers, cohesin was found to both promote and counteract their interactions 64, 80. Finally, in the case of E–P loops, extrusion likely cooperates with compartmentalisation and contributes to loop formation, especially for long-range enhancers 23•, 29, 36, 38••.

Taken together, this suggests that while extrusion may prevent hyper-compartmentalisation in some cases, it may actually promote the formation of compartmentalised chromatin in others. How these two mechanisms interact will depend on loop type, heterogeneity in genomic context, chromatin-bound proteins, nuclear localisation and distance between loci, and will ultimately shape the three-dimensional (3D) genome. In addition, loop extrusion and compartmentalisation are likely to be influenced by other nuclear processes involved in chromatin organisation, such as chromatin tethering to the nuclear lamina, nuclear body formation and protein-RNA interactions.

Further dissection of the effects of loop extrusion and compartmentalisation on chromatin loops requires methods to selectively manipulate these processes. Although such approaches are available for rapid and reversible modulation of loop extrusion components [81], methods for equally precise control of compartmentalisation processes are still in their infancy. Such techniques, combined with recent advances in chromosome conformation capture methods, live-cell imaging and biophysical simulations, would allow scientists to study 3D genome organisation in unprecedented detail. Together, this will provide further insights into the interplay between different chromatin folding mechanisms and help identify novel looping factors.

## Declaration of Competing Interest

The authors declare no conflict of interest.

## Data Availability

No data were used for the research described in the article.
